# Hodgkin lymphoma: flow me?

**DOI:** 10.1186/1742-6413-2-13

**Published:** 2005-09-08

**Authors:** Michael W Beaty, Kim R Geisinger

**Affiliations:** 1 Wake Forest University School of Medicine Department of Pathology Medical Center Boulevard Winston-Salem, North Carolina 27157-1072 USA

## Abstract

Combining fine needle aspirate cytology with flow cytometry immunophenotyping for the rapid diagnosis of lymphoproliferative lesions is commonplace practice in many institutions. Yet, a definitive diagnosis of Hodgkin lymphoma in many cases remains elusive, requiring subsequent tissue biopsy confirmation. In this issue of CytoJournal, Hernandez et al explore the potential role of using the increased CD4/CD8 T-cell ratio in lymph node fine needle aspiration specimens as a specific feature in diagnosing Hodgkin lymphoma. CD4/CD8 T-cell ratio comparisons are made with cytomorphologic diagnoses of reactive, atypical, non-Hodgkin lymphoma, and Hodgkin lymphoma cases.

## 

Fine needle aspiration (FNA) cytomorphology in conjunction with flow cytometric (FC) immunophenotyping has become a reliable and accurate method for the diagnosis and classification of many lymphoproliferative disorders. The combined modality approach often allows for confirmation of diagnosis and subtyping of lymphoma, and in many cases obviates the necessity for a more invasive open biopsy for many patients with lymphadenopathy [1]. This is especially true in suspected cases of non-Hodgkin lymphomas (NHL). However, even with the collaborative expertise that we and others routinely employ to reach a consensus diagnosis between the cytopathologist interpreting the FNA material and the hematopathologist signing-out the FC data, the diagnosis of Hodgkin lymphoma (HL) through this method remains difficult. A FNA diagnosis of HL based purely on cytomorphology is often followed by tissue biopsy [2].

One of the major difficulties encountered with combined FNA/FC diagnosis of HL is identifying sufficient numbers of the neoplastic cells for subsequent phenotypic analysis. In most instances, too few malignant cells for FC can be obtained from the tumors, either by FNA or biopsy material. This is due, in large part, to the cellular makeup of HL, in which the inflammatory background cells outnumber the malignant RS cells and variants by 10–100 fold. In addition, the neoplastic cells exhibit high cellular fragility, and are associated with a fibrous stroma interfering with cellular dispersion. This has led to Herculean gating efforts to isolate the RS cells and phenotype them with CD30 and CD15 [3,4]. Many other studies have looked at the components of the normal background T-lymphocyte populations present in HL, where it has long been known that there is a relative preponderance of CD4-positive T-cells, with an increased helper/cytotoxic CD4/CD8 ratio by immunohistochemistry [5-7]. We know that the RS cells and variants within the lymph node affect the surrounding cells, which results in alterations of the proportion between T and B lymphocytes and the activation of these cells, and that the relative proportion of the background lymphocytes differs depending upon the histologic subtype of the HL [8]. While the nature and composition of the background reactive lymphocytes is helping to increase our basic understanding of the pathogenesis of the disease, it can hardly be used as a specific diagnostic feature.

In the current issue of Cytojournal, Hernandez et al compare 85 cases of combined FNA/FC lymph nodes with an increased CD4/CD8 ratio (>4), and demonstrate no definitive quantitative differences in T-lymphocyte distribution between HL, NHL, and benign reactive lymph nodes. They report that the average CD4/CD8 ratio was essentially identical in reactive, HL, and NHL cases. This should not be surprising given the extraordinary variation in lymphocyte components in many lymphoproliferative processes. T-cell rich large B-cell lymphoma and nodular lymphocyte predominant HL are just two tumors which quickly come to mind. Both of these tumors can morphologically mimic classical HL particularly on FNA material, with only a minor population of malignant RS-like cells within a sea of reactive small lymphocytes. Phenotypic studies confirm that CD4-positive cells may exceed the CD8-positive background lymphocytes [10,11].

In a recent report by Ravoet, et al, FC was combined with lymph node biopsy to help discriminate HL, NHL, carcinoma, and reactive diagnoses [4]. Median CD4/CD8 ratios in HL were 5.0 (range 1.6–10.4), which was similar to that seen in granulomatous lymph nodes (median 3.2, range 1.5–11.8), and reactive hyperplasia (median 2.71, range 0.4–18.6). They concluded that given the large scattering of the CD4/CD8 ratios within each group, the use of the CD4/CD8 ratio is not recommended in clinical practice in order to diagnosis HL.

A quick review over the past year of lymph node FNAs performed at our institution reveals a similar wide scattering of ratios. We identified 6 cases of FNA suspected HL with a median CD4/CD8 ratio of 9.7 (range 0.3–42.5). Subsequent nodal biopsies confirmed HL in all cases. The CD4/CD8 ratio was less than 4 in half of these cases. Interestingly, one patient with an HIV-associated HL predictably had a reverse ratio, another potential pitfall. Equally important though were the six additional cases of suspected HL by FNA in which the specimens received for FC were inadequate, containing too few cells to phenotype. Thus, we rely exclusively on the morphologic features present within the FNA smears, combined with confirmatory immunohistochemical studies performed on cell block material to diagnosis HL with confidence. Otherwise, a nodal biopsy is and should be recommended [1].

Integration of clinical and biological features is one of the basic tenets of the WHO classification of lymphoproliferative disorders. While combined FNA/FC is extremely useful in the diagnosis and subclassification of some lymphoproliferative disorders, it is not without limitation and is not the end of the diagnostic line. Until we know more about the characteristics of the T-cell milieu of HL and can point to a more specific phenotype which can be detected by FC, we must continue to rely on other ancillary tools for a definitive HL diagnosis. The work of Hernandez et al is important to remind us that an increased CD4/CD8 ratio is only one phenotypic clue present in HL, and a non-specific clue at that.

## Note

Corresponding article: Hernandez O, Oweity T, Ibrahim S: **Is an Increase in CD4/CD8 T-cell ratio in lymph node fine needle aspiration helpful for diagnosing Hodgkin lymphoma? **Cytojournal 2005, 2:14 [9].
